# Discovery through Machine Learning and Preclinical Validation of Novel Anti-Diabetic Peptides

**DOI:** 10.3390/biomedicines9030276

**Published:** 2021-03-09

**Authors:** Rory Casey, Alessandro Adelfio, Martin Connolly, Audrey Wall, Ian Holyer, Nora Khaldi

**Affiliations:** Nuritas Ltd., Joshua Dawson House, D02 RY95 Dublin, Ireland; casey.rory@nuritas.com (R.C.); adelfio.alessandro@nuritas.com (A.A.); connolly.martin@nuritas.com (M.C.); info@nuritas.com (I.H.); n.khaldi@Nuritas.com (N.K.)

**Keywords:** drug discovery, peptide, type 2 diabetes, machine learning

## Abstract

While there have been significant advances in drug discovery for diabetes mellitus over the past couple of decades, there is an opportunity and need for improved therapies. While type 2 diabetic patients better manage their illness, many of the therapeutics in this area are peptide hormones with lengthy sequences and a molecular structure that makes them challenging and expensive to produce. Using machine learning, we present novel anti-diabetic peptides which are less than 16 amino acids in length, distinct from human signalling peptides. We validate the capacity of these peptides to stimulate glucose uptake and Glucose transporter type 4 (GLUT4) translocation in vitro. In obese insulin-resistant mice, predicted peptides significantly lower plasma glucose, reduce glycated haemoglobin and even improve hepatic steatosis when compared to treatments currently in use in a clinical setting. These unoptimised, linear peptides represent promising candidates for blood glucose regulation which require further evaluation. Further, this indicates that perhaps we have overlooked the class of natural short linear peptides, which usually come with an excellent safety profile, as therapeutic modalities.

## 1. Introduction

Type 2 diabetes mellitus (T2DM) is a chronic condition which accounts for over 90% of all diabetes mellitus (DM) incidences. In T2DM, cells fail to respond to the hormone insulin, or a relative lack of insulin is produced by the beta cells of the pancreas, which normally allows glucose to enter cells from the blood, reducing blood glucose levels. This condition has long-term implications, affecting several organs in the body, such as nephropathy of the kidney and retina, and hepatic steatosis, all of which contribute to poor quality of life and a high burden on healthcare systems [1]. Major risk factors for T2DM include obesity, lack of exercise and sedentary lifestyle, all of which are increasingly common in the West. Currently, the global incidence of DM continues to grow at an inexorable rate, currently affecting over 450 million people, and is expected to afflict almost 700 million by 2045 [2]. While DM drug discovery has seen some important advances over the last two decades [3,4], in light of such widespread disease prevalence, there is an evident and urgent need for novel, effective anti-diabetic treatments that have improved safety profiles and are well tolerated for chronic use in the DM population [5,6].

Pharmacological interventions for T2DM include metformin, a small-molecule drug known to work via several mechanisms, including AMP-activated protein kinase (AMPK) [7] and mitochondrial activity [8,9]; it is a well-established first line treatment for T2DM, both as a monotherapy and in combination with other medications, and has been routinely shown to have glucose-lowering effects [10]. Thiazolidinediones are cyclic compounds that act as ligands to peroxisome proliferator-activated receptors (PPARs), reducing circulating fatty acids and increasing the expression of glucose transporter Glucose transporter type 4 (GLUT4), allowing cells to take up more glucose from the blood for energy, reducing blood glucose levels [11,12]. More recently, sodium glucose transporter 2 (SGLT2) inhibitors have been developed, which work to prevent the action of the transporters in the proximal tubule of the kidney from reabsorbing glucose to the body, thereby allowing excretion and reducing blood glucose [13]. Indeed, SGLT2 inhibitors are often used in combinational therapy with other medications to help manage the complex pathophysiology of T2DM [14].

An important and efficacious group of T2DM therapeutics includes a group of peptides such as insulin analogues, and, more recently, incretin mimetics, acting as either agonists (e.g., Glucagon-like peptide-1 (GLP-1) and glucose-dependent insulinotropic polypeptide (GIP) analogues) or antagonists (e.g., GLP-1 receptor antagonists) of endogenous human hormones, with modifications [15]. These therapeutics form a significant number of the modern anti-diabetic drugs and also those in the development pipeline [16]. This group has been shown to suppress glucagon and hepatic glucose production, slow gastric emptying and reduce appetite [17], with two approved members of the class, Exenatide and Liraglutide, having long-term weight loss effects on patients over a 1–2-year period [18]. Both Exenatide and Liraglutide are relatively shorter compared to 51-amino-acid-long insulin, at 39 amino acids and 32 amino acids in length, respectively. Both medications are administered as subcutaneous injectables and havepresented with some adverse effects, including vomiting and nausea [19,20].

An alternative class of peptides which may offer good safety profiles and toleration for chronic use, but have been largely underexplored, are short linear peptides with few modifications. Linear endogenous and synthetic peptides have been shown to be capable of modulating intracellular signalling, without modifications [21]. As such, these advantages position this class as an attractive addition to the diabetes armamentarium. Indeed, peptides can be highly selective, having multiple points of contact with their target, which may result in decreased side effects and toxicity [22]. Furthermore, as they comprise amino acids, peptides are easily metabolised over time, thereby avoiding the tolerance issues that can be associated with chronic administration of many drugs [22,23]. A possible advantage of short linear peptides includes lower manufacturing costs and offers a flexible base for modifications [24,25]. However, presently, a major drawback of these peptides is that they are readily broken down during gastrointestinal digestion (GID); therefore, issues with low bioavailability via oral administration remain problematic [26]. This has resulted in a turn towards developing optimised peptides to enhance therapeutic properties, such as cyclisation [27], although there are instances where anti-cancer linear peptides outperform their cyclic counterparts [25], indicating that this class of peptides should not be so readily dismissed.

Biology is an extremely data-rich discipline owing to various “omics” technologies producing increasingly larger volumes of heterogenous data [28]. In recent years, integration of such data has facilitated a greater understanding of the molecular basis of disease [29], but with continued escalation in terms of scale and complexity, human-directed interpretation is rendered increasingly impractical [27,30]. When considering peptides, deciphering scale and complexity becomes a major hurdle; for example, proteins can be broken down into peptides at a rate of 36 million per minute [21]. However, Artificial Intelligence (AI) and deep learning techniques are perfectly primed to extract previously indecipherable knowledge from disparate biological data streams; as such, machine learning is increasingly seen as a discovery tool in life science, with bioactive peptides being successfully predicted in the areas of inflammation and skin aging [31,32,33,34]. Here, similar machine learning methods were employed to identify a short linear novel peptide therapeutics for use in T2DM. The goal of this project was to identify (a) peptide(s) which could modulate an effect on blood glucose levels, GLUT4 expression and/or glycated haemoglobin (HbA1c) levels, while being both non-toxic and showing no off-target effects. The peptide candidates were validated in both in vitro and in vivo assays to ensure these properties.

## 2. Materials and Methods

### 2.1. Cell Line

Human skeletal muscle cells (HSkMCs; Cell Applications Inc., San Diego, CA, USA) were cultured at 37 °C, 5% CO_2_ in HSkMC growth medium (Cell Applications Inc., San Diego, CA, USA). HSkMCs cultured for, at most, 10 passages were used for all experiments described.

### 2.2. Animals

All animal procedures were carried out in accordance with Institutional Animal Care and Use (IACUC) guidelines in an Association for Assessment and Accreditation of Laboratory Animal Care International-accredited facility. Ethical approval was granted by the International Association of Religious Freedom (IARF #:MLR-101, IARF #:MLR-115) Studies were performed with 12-week-old male KK.Cg-A^y^/J (KK-A^y^) mice obtained from the Jackson Laboratory, which were randomly assigned to treatment groups according to baseline fasting blood glucose (IARF #: MLR-101, 1 May 2018). Mice were housed with no more than 4 per cage on a 12-h light/dark cycle with ad libitum access to standard rodent chow and water. Mice were subcutaneously (sc) administered either indicated doses of peptide or saline (untreated vehicle control) once daily for 2 weeks. Bodyweight was measured at baseline and once per week thereafter. Fasting (overnight) blood glucose was measured at baseline, day 5, day 7 and day 13 of dosing (cohort 1). In a second, independent in vivo study (IARF #: MLR-151; 13 January 2020), mice (n = 11/group) were sc administered either indicated dose of peptide, Liraglutide or saline for 6 weeks. Glycated haemoglobin was measured at 6 weeks, and liver sectioning via microtome, staining with haematoxylin and eosin (H&E) and scoring were performed on snap-frozen tissues.

### 2.3. Prediction Workflow

**Figure 1:**
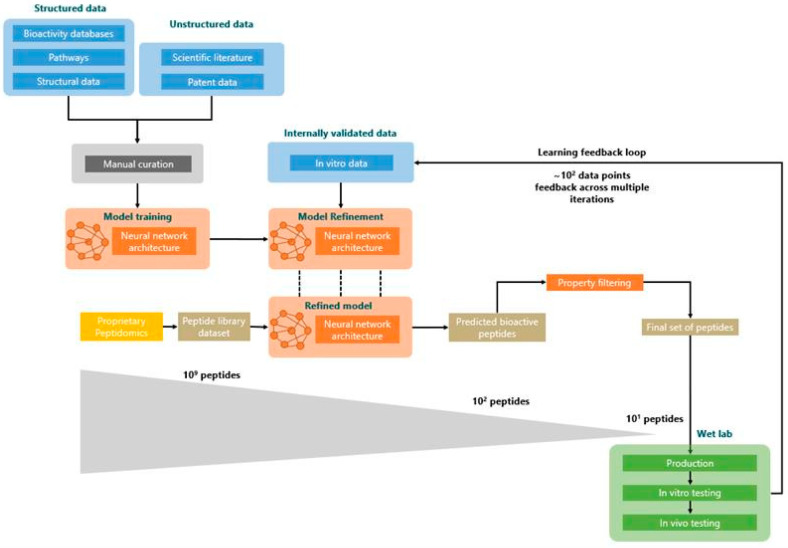
Peptide prediction. Workflow for predictive models adapted from Corrochano et al., 2021 [35].

A similar predictive model to that used by Kennedy et al., 2020 [33] was utilised here; briefly, the model was developed using an ensemble of neural networks. To build the training set for the model, we used structured data from public databases (bioactivity annotations, biological pathways and structural annotations) and unstructured data extracted from peer-reviewed scientific papers and patents (Figure 1). Initial descriptors used to query these data sources were “diabetes”, “blood glucose regulation” and “GLUT4”. A combination of graph-based techniques and manual curation was used to process the structured data, while, concurrently, Natural Language Processing (NLP) techniques, such as word and sentence embedding and named entity recognition, were applied to the unstructured data. The high-level information extracted was assembled and formatted into a bespoke peptide representation format.

The resulting dataset of peptides with a known effect on blood glucose regulation was used to train our predictive architecture for bioactivity in fold cross-validation. The fully trained model was used to predict novel peptides’ glucose uptake efficacy from a large input set of peptide sequences.

Additional testing and refinement of the predictive architecture was achieved by incorporating a predict–test–refine loop. The predict–test–refine loop is an example of active learning. Uncertainty sampling was performed where peptides that the model was least certain as to what their activity should be were selected for experimental testing. This strategy was mixed with the selection of the best predicted peptides, which were tested concurrently. Uncertainty sampling was prevalent in the first iterations and progressively reduced to be completely replaced by the identification of the most promising peptides in the latest iteration. The results of in vitro testing were additionally integrated into the model to bias it towards the prediction of peptides with specific GLUT4 translocation activity. In this framework, a set of peptides, which the model found most difficult to classify (i.e., those with an efficacy prediction close to 50%), were selected for experimental testing in vitro, with resultant data being fed back to the predictive model. This predict–test–refine loop was completed three times. Across the multiple iterations, in vitro activity was measured in 74% of cases, where peptides tested in glucose regulation assays would show what was internally classified as “medium to high activity”. A similar active learning paradigm was used in Kennedy et al., 2020 [33]. In that case, a lower ratio of in vitro activity was measured across the multiple lab tests, with only 40% of peptides tested in extra cellular matrix development assays displaying “medium to high activity”.

At the end of the refinement process, a set of 10^9^ peptides was fed into the model, which returned an output set of 10^2^ peptides classed as “active.”

Next, a further collection of internally built tools was used to filter out sequences exhibiting undesirable properties. To narrow down the number of relevant peptides to be tested at each iteration (from hundreds to tens), predicted peptides were ranked using different internal predictive models. Specifically, peptides predicted as cell-penetrant, stable in blood and not toxic were prioritised. Additionally, other filters were applied, removing peptides with an odd number of cysteines or with sequence longer than 30 residues, to facilitate synthesis. Finally, all peptides exhibiting high homology against peptides contained known to have a role in glucose regulation were discarded. This final stage ultimately left a set of 5 distinct, novel peptides suitable for experimental validation.

### 2.4. Homology Searching and Synthesis of Predicted Peptides

To determine the true novelty of our 5 predicted peptides, we measured their homology to (1) each other and (2) to analogues or antagonists of endogenous human hormones. All searches were performed using BLASTP from the BLAST+ (BLAST+, v2.2.31) suite of programs using the following parameters: word size = 2; matrix = PAM30, E-value = 10,000. Predicted peptides were chemically synthesised by GenScript Corporation (Piscataway, New Jersey, United States). For all peptides screened at initial stages, theoretical molecular weight (MW) was checked, and all were confirmed to have HPLC purity of ≥95.0%. pep_1E99R5 had a theoretical MW of 1270.48 with a HPLC purity of ≥98.0%.

### 2.5. Glucose Uptake Assay

HSkMCs were plated on collagen-coated 96-well plates (1 × 10^4^/well) and allowed to adhere overnight at 37 °C, 5% CO_2_ in 100 µL of HSkMC growth medium. The medium was then changed to HSkMC differentiation medium (Promocell, Heidelberg, Germany) and cells were allowed to differentiate for 7 days, with fresh medium added every 2 days. The day prior to the experimentation, cells were starved overnight in basal medium. Glucose uptake was measured using a glucose uptake assay kit (Abcam, Cambridge, MA, USA) as per the manufacturer’s instructions. Briefly, cells were rinsed three times in Dulbecco’s phosphate-buffered saline (DPBS; Lonza, Basel, Switzerland) and then starved of glucose by incubating with 100 µL of Krebs-Ringer-Phosphate-Hepes (KRPH) buffer for 40 min at 37 °C. KRPH buffer was made with 20 mM HEPES, 5 mM monopotassium phosphate, 1 mM magnesium sulphate, 1mM calcium chloride, 136 mM sodium chloride, 4.7 mM potassium chloride, adjusted to pH 7.4, all compounds were purchased from Sigma-Aldrich (St. Louis, MO, USA). Cells were treated with HSkMC basal medium containing 0.5 µg/mL (~0.4 µM) of peptide or 1 µM of human insulin solution (Sigma-Aldrich, St. Louis, MO, USA) for 20 min, followed by incubation with 10 µL of 2-deoxyglucose (2-DG; Glucose uptake assay kit; Abcam, Cambridge, UK) for 20 min at 37 °C. Subsequently, cells were washed 3 times with PBS and lysed with 80 µL of extraction buffer, after which cell lysates were freeze–thawed once before heating at 85 °C for 40 min. Following cooling on ice for 5 min, lysates were neutralised by adding 10 µL of neutralisation buffer and then diluted with assay buffer to a total volume of 50 µL (5 µL lysate + 45 µL assay buffer). After two amplification reactions, absorbance of the samples was measured at 412 nm with a microplate spectrophotometer.

### 2.6. GLUT4 Translocation Assay

HSkMCs aliquots (2 × 10^5^/well) were seeded in muscle growth medium in collagen-coated 6-well plates and differentiated in differentiation medium for 7 days. The day prior to the experimentation, cells were starved overnight in basal medium. The cells were treated with peptide (0.5 µg/mL (~0.4 µM)) or 1 µM human insulin solution for 20 min. Membrane proteins were then solubilised and isolated from cytosolic proteins using a Mem-PER™ Plus Membrane Protein Extraction Kit (ThermoFisher, Waltham, Mass, USA) as per the manufacturer’s instructions. A 1-µM treatment with insulin to investigate glucose uptake and the associated downstream signalling is widely used in the literature, in both rat and human cell line models [36,37]. GLUT4 concentration was subsequently measured via a commercially available Human GLUT4 Sandwich ELISA kit (Abbexa, Cambridge, UK). Briefly, 100 µL of standard, blank or sample was loaded into individual wells of a 96-well plate and incubated at 37 °C for 2 h. Liquid was then aspirated and 100 µL of detection reagent A was added for 1 h at 37 °C. Subsequently, liquid was aspirated from each well, which was then washed 3 times using 350 µL of wash buffer before detection reagent B was added for 30 min at 37 °C. Wells were washed 5 times, as before, following which 90 µL of 3,3',5,5'-Tetramethylbenzidine substrate was added to each well and incubated at 37 °C for 10 min while protecting from light. Then, 50 µL of Stop solution was then added, after which the optical density of the sample was determined using a microplate reader (SpectraMax M3, Molecular Devices, Sunnyvale, CA 94089, USA) set to 450 nm.

### 2.7. Microarrays

HSkMCs aliquots (2 × 10^5^/well) were seeded in muscle growth medium in collagen-coated 6-well plates and differentiated in differentiation medium for 7 days. The day prior to the experimentation, cells were starved overnight in basal medium. The cells were treated, in triplicate, with peptide (0.5 µg/mL (~0.4 µM)) or 1 µM or human insulin solution for 20 min, at which point the treatment medium was removed and the cells were scraped in 1 mL of PBS. The cell suspension was pelleted in a microcentrifuge at 1500 rpm for 5 min, and the supernatant was removed. The cells were immediately flash-frozen in liquid nitrogen and transferred to a −80 °C freezer for storage prior to RNA extraction. RNA was extracted in accordance with standard operating procedures for RNA extraction from tissue/cell pellets using a RNeasy mini kit (Qiagen, Manchester, UK). RNA quality and integrity were determined via bioanalyser. For each microarray experiment, 100 ng of total RNA was used. To study the whole genome expression with a comprehensive coverage of genes and transcripts, 26,083 Entrez Genes and 30,606 lncRNA, SurePrint G3 Human Gene Expression v3, 8 × 60 K Microarrays (Agilent, Santa Clara, CA, USA) were used. Microarray gene expression experiment was performed according to the manufacturer’s protocol (One-Color Microarray-Based Gene Expression Analysis—Low Input Quick Amp Labeling v6.9). After the experiment, the arrays were scanned by SureScan Microarray Scanner (Agilent, Santa Clara, CA, USA) and data were extracted using Feature Extraction Software (Agilent, Santa Clara, CA, USA). The samples were prepared for array hybridisation according to the manufacturer’s protocol. Briefly, labelled cRNA was hybridised to the microarray for 16 h before the array slides were washed and scanned using an Agilent G2565CA Microarray Scanner System.

### 2.8. Transcriptomics and Pathway Enrichment

Microarray data analysis was conducted using the R Bioconductor package limma [38]. Intensities were background corrected using the normexp method with an offset of 50 [39]. Quantile normalisation was then applied. Due to the use of several arrays, subsequent batch effect was identified and removed using ComBat (sva R package) [40]. Each treated group was compared to the negative untreated control using linear modelling, from which an empirical Bayesian analysis was then performed using the function ebayes from limma.

To initiate the pathway enrichment analysis, significant gene lists were filtered using raw *p*-value < 0.01 and fold-change > 1.3. Enrichment was performed on Kyoto Encyclopedia of Genes and Genomes (KEGG) pathways using a hypergeometric test. Pathways were considered enriched at raw *p*-value < 0.01. Enrichment was assessed for each treatment insulin and pep_1E99R5, respectively, considering up- and downregulated gene lists. All analysis was performed internally and by third-party Fios Genomics Ltd. (Edinburgh, UK).

### 2.9. Statistical Analyses

All data are presented as mean ± SEM. Replicate numbers for each experiment are indicated in figure legends. Results of in vitro experiments were assessed by one-way ANOVA followed by Dunnett’s post-hoc test. Comparisons between different peptide treated and untreated KK-A^y^ groups were assessed by one-way ANOVA followed by Dunnett’s post-hoc test. Statistical significance was defined as *p* < 0.05. Graphs were generated using the “ggplot2” R package [41].

## 3. Results

### 3.1. Novel Peptide Prediction and Validation

Using a machine learning approach similar to Kennedy et al., 2020 [33], one hundred peptide candidates were predicted as potentially possessing anti-diabetic functionality via blood glucose regulatory activity. This set of peptides was further refined using a collection of tools to filter out the sequences with undesirable physiochemical properties. Taking into account the significant costs involved in peptide drug manufacturing [42], all predicted peptides were to be less than 20 amino acids in length and linear with no major structures. Ultimately, this resulted in a final set of five peptides that interestingly exhibited no homology to each other or to any known patented or published bioactive peptides. These peptides are referred to hereafter as pep_1E99R5, pep_37MB3O, pep_ANUT7B, pep_RTE62G and pep_QT5XGQ. To validate the bioactivity of these predicted peptides, an in vitro glucose uptake method was employed.

**Figure 2:**
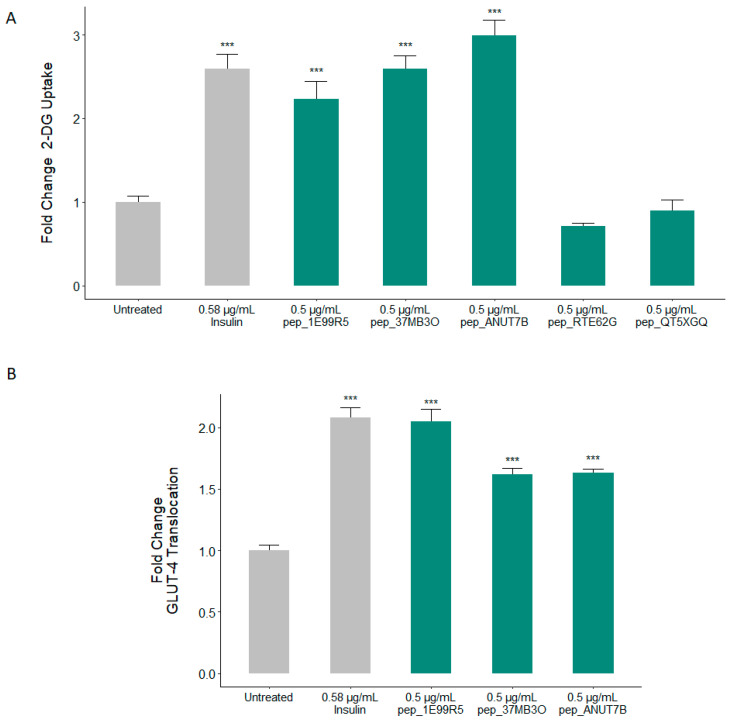
In vitro validation of predicted peptides as potent stimulators of glucose uptake and Glucose transporter type 4 (GLUT4) translocation. Skeletal muscle cells were stimulated with either insulin or predicted natural peptides at the indicated concentrations for 15 min. (**A**) The effect on glucose uptake is measured, while in (**B**), the extent of GLUT4 translocation is assessed (one-way ANOVA analysis with Dunnett’s test; *** *p* < 0.001; data presented are the mean ± SEM of at least 3 independent replicates).

Insulin-stimulated uptake of blood glucose by skeletal muscle plays a fundamental role in the maintenance of glucose homeostasis, accounting for 75% of glucose utilisation in the body [43]. Accordingly, the predicted bioactivity of our predicted peptides was first validated in a cell-based glucose uptake assay, where insulin was used as a positive control. Three predicted peptides, pep_1E99R5, pep_37MB3O and pep_ANUT7B, demonstrated the ability to significantly increase glucose uptake in human skeletal muscle cells (HSkMCs), with pep_37MB3O and pep_ANUT7B displaying a stronger effect than that of insulin (Figure 2A). No significant glucose uptake activity was reported for pep_RTE62G and pep_QT5XGQ; thus, these peptides were not progressed further in our in vitro and in vivo validation studies. Cumulatively, these results indicate an in vitro validation success rate of 60% for the predictive model and suggest that these sequences should be further examined in relevant models. Of note, a more comprehensive absorption, distribution, metabolism, and excretion (ADMET) prediction could be incorporated for further development of the presented peptides.

The key mediator of glucose uptake into skeletal muscle cells is the protein GLUT4 [44]. Skeletal muscle accounts for the majority of glucose uptake in the body [45]. While glucose uptake efficacy was used as an initial experimental validation of our predicted peptides, the ultimate objective of the predictor was to identify peptides capable of stimulating GLUT4 translocation to the plasma membrane, which is known to be decreased in type 2 diabetics [46]. Indeed, interventions to promote the expression and translocation of GLUT4 in appropriate cells such as skeletal muscle fibres are of clear benefit to those with T2DM [47]. The HSkMC model used for this study allows for measurement of GLUT4 translocation, which, in turn, allows for a specific molecular mechanism to be identified as the cause of these peptides’ ability to modulate glucose levels. Accordingly, we evaluated the ability of these peptides to initiate GLUT4 translocation in HSkMCs. At a test dose of 0.5 µg/mL, we found that all three predicted peptides stimulated a highly significant increase in GLUT4 translocation (Figure 2B). Of the three positively predicted peptides, pep_1E99R5 is seen to demonstrate the most potent effect, eliciting an approximately equivalent response to insulin (Figure 2B).

### 3.2. Validation of Peptides in a Diabetic Mouse Model

**Figure 3:**
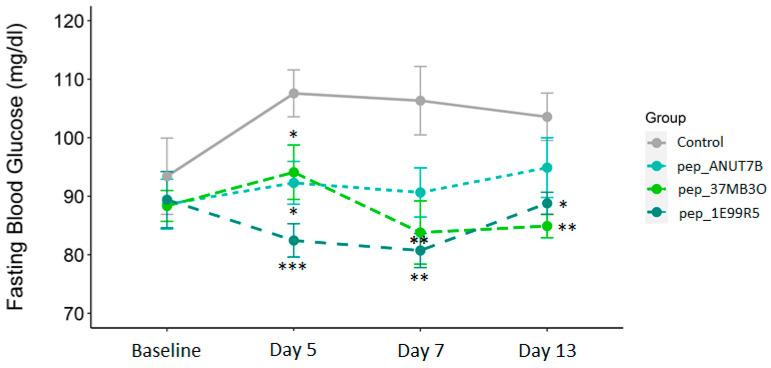
Predicted peptides in a mouse model of type 2 diabetes. Fasting glucose levels (overnight) of all mice were measured as indicated. Data are mean ± SEM (*n* = 8 per group; aged 12 weeks at baseline) and analysed by Dunnett’s test to compare the differences between the peptide treatment groups and the vehicle control group (* *p* < 0.05 ** *p* < 0.01 *** *p* < 0.001).

The therapeutic potential of our in-vitro-validated anti-diabetic peptides was evaluated in the KK-A^y^ model of obese insulin-resistant DM. In KK-A^y^ mice, fasting blood glucose and HbA1c levels are elevated, and when fed a normal diet, obesity and DM are observed by 12 weeks. Peptides pep_37MB3O, pep_1E99R5 and pep_ANUT7B at 127 mg/kg (100 µM) or vehicle were dosed subcutaneously (sc) once daily for 14 days. All animals tolerated treatment well and body weight did not change per treatment throughout the study (Figure S1). At day 5 post-baseline, all three predicted peptides significantly reduced fasting blood glucose compared to the vehicle control group (Figure 3), with pep_1E99R5 demonstrating the most significant reduction. The effect of pep_ANUT7B was tempered by day 7 (*p* = 0.07) and day 13, with pep_1E99R5 and pep_37MB3O both exhibiting a significant reduction in fasting blood glucose at these time points. However, the effect of pep_1E99R5 was slightly reduced at day 13 (Figure 3), a trend that is in line with what is observed for known anti-diabetic therapies in several animal studies [48,49]. While pep_1E99R5 and pep_37MB3O’s effects were maintained throughout the study period, pep_1E99R5 treatment resulted in the most potent effects on fasting blood glucose levels in vivo; therefore, this peptide was chosen for a longer in vivo study to measure HbA1c.

**Figure 4:**
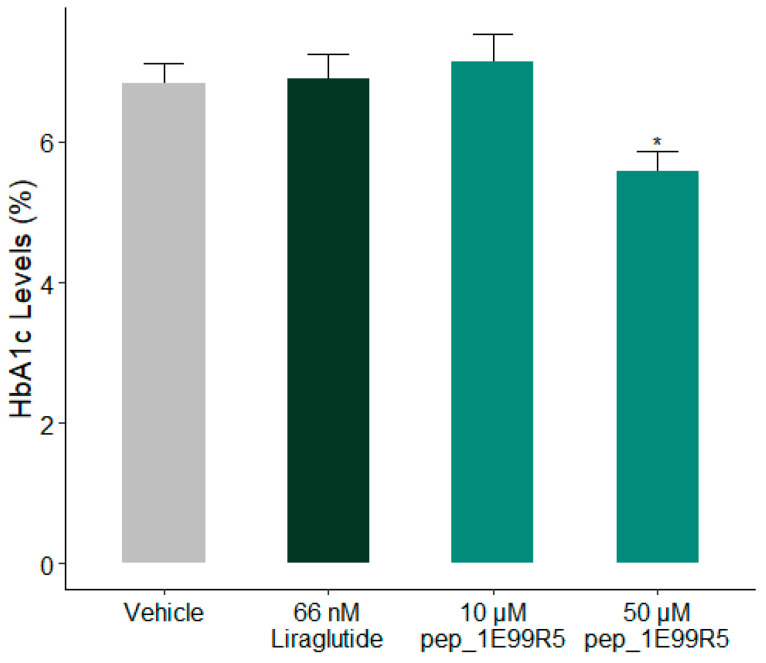
Effect of pep_1E99R5 on HbA1c levels. HbA1c levels (%) of all mice were measured as indicated. Data are mean ± SEM (n = 11 per group; aged 12 weeks at baseline) and analysed by Dunnett’s test to compare the differences between peptide treatment groups, Liraglutide group and vehicle control (* *p* < 0.05).

As a decrease of >1% HbA1c is considered to be of significant clinical benefit [50], the effects of pep_ 1E99R5 on HbA1c % were measured in a trial over 6 weeks. In KK-A^y^ mice, pep_1E99R5 (12.7 mg/kg (10 µM) or 63.5 mg/kg (50 µM)) was administered daily via sc injection and compared to control groups of vehicle or Liraglutide (250 µg/kg), previously shown to have a positive effect on HbA1c [51]. HbA1c levels are a long-term indicator of blood glucose regulation [52]; previous studies have shown significant treatment effects on HbA1c at 8 weeks in similar KK-A^y^ models [53]. However, Liraglutide treatment did not significantly reduce HbA1c percentage compared to vehicle control following 6 weeks of treatment (Figure 4); it is possible that a significant drop in HbA1c levels would have been recorded if the study duration were extended. In contrast, pep_1E99R5 (63.5 mg/kg)-treated mice showed a significant reduction of approximately 1.3% in HbA1c compared to vehicle control, suggesting a sustained effect of pep_1E99R5 in the animals. No effect was noted in the 10-µM-treated mice, which suggests a dose-dependent effect of the peptide.

A common complication associated with T2DM is hepatic steatosis (HS), a condition in which fatty deposits accumulate in the liver, affecting up to 75% of T2DM patients, often leading to non-alcohol fatty liver disease (NAFLD) [54]. Moreover, HS among the prediabetic population is considered to be a predictor of conversion to DM [55]. In a study by Fiorentino et al., a subset of HbA1c-defined prediabetic individuals with 1-h postload glucose ≥155 mg/dL were at higher risk of developing HS [56]. Furthermore, the prevalence of prediabetes and DM was found to be six-fold higher in NAFLD patients compared to healthy controls. [57] Therefore, a decrease in HS, alongside the HbA1c decrease, would be of significant clinical interest.

**Figure 5:**
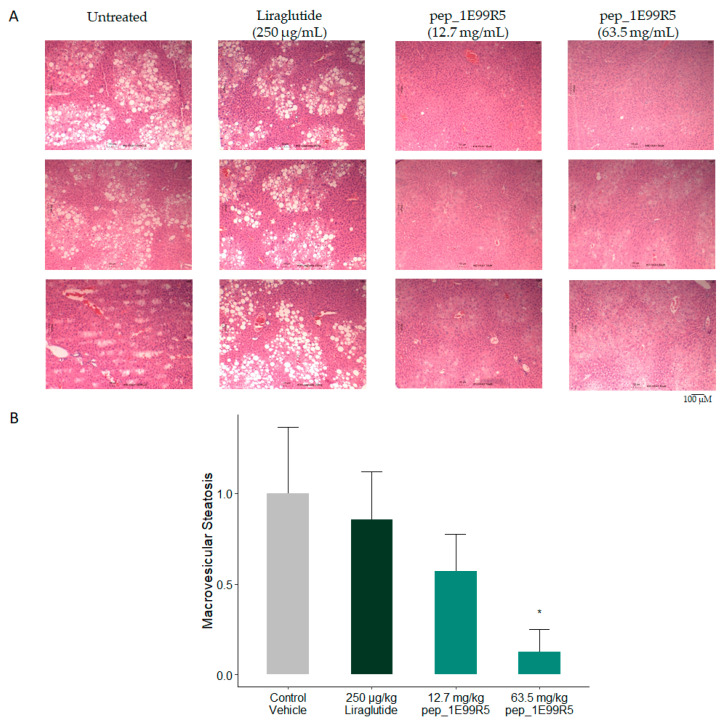
Effect of pep_1E99R5 on hepatic steatosis. (**A**) Livers of all mice were excised and sectioned before staining with H&E. Images were taken using a Motic BA310E trinocular compound microscope at 10x magnification. Histological non-alcohol fatty liver disease (NAFLD) scoring was performed by an independent third-party reviewer. (**B**) Features were scored according to a murine liver scoring system devised by Liang et al., 2014. Data are mean ± SEM (*n* = 6 per group; aged 12 weeks at baseline) and analysed by Dunnett’s test to compare the differences between the two peptide treatment groups and vehicle control and Liraglutide groups (* *p* < 0.05).

Consequently, levels of hepatic steatosis (HS) in the mice were measured via sectioning and H&E staining. Histological NAFLD scoring was performed by an independent third-party reviewer. Features were scored according to a murine liver scoring system devised by [58] (Figure 5A). Vehicle control and Liraglutide-treated mice exhibited signs of NAFLD; however, these were reduced significantly in 50-µM pep_1E99R5 and trended to a decrease in 10-µM pep_1E99R5 treatment, suggesting a dose-dependent effect (Figure 5B).

### 3.3. Characteristics of Predicted Peptides

**Figure 6:**
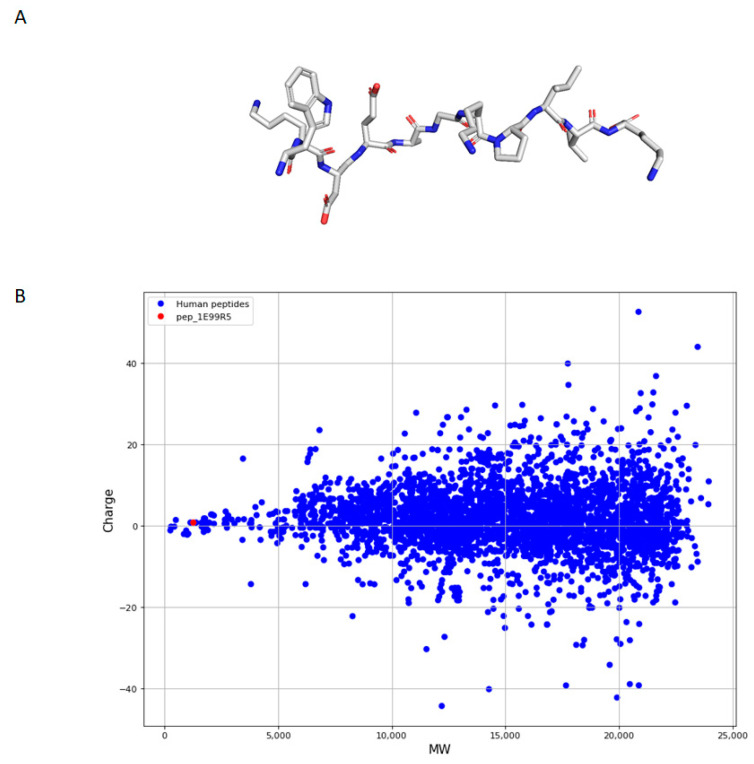
Linear representation of pep_1E99R5 and molecular weight and charge dispersion of pep_1E99R5 compared to endogenous human peptides. (**A**) Representation of the linear structure for pep_1E99R5 generated using PyMol, Version 2.3.5, Schrödinger, LLC (stick visualisation). (**B**) Dispersion of molecular weight and charge of human endogenous peptides (blue) and pep_1E99R5 (red). The human peptides were retrieved from UniprotKB (https://www.uniprot.org/statistics/Swiss-Prot (Accession date: 8 March 2021)) and filtered with a threshold of 200 amino acids. The average molecular weight of the human peptides is 14.4 kDa (Q1 = 11.3 kDa, Q3 = 18.1 kDa) while pep_1E99R5 is 1.3 kDa. The average charge of the human peptides is +2.9 (Q1 = −1, Q3 = +7) while pep_1E99R5′s charge is +1.

As short linear peptides offer an intriguing therapeutic option due to decreased side effects and toxicity [22], the predictive model focused on peptides <20 amino acids (AA) in length. Of note, pep_1E99R5 consists of 11 AA, WKDEAGKPLVK, with no major structures (Table 1; Figure 6A). While this specific sequence of amino acids was predicted due to desirable features identified through the predictive model, further work is being carried out on derivatives of pep_1E99R5 to assess bioactivity retention. Apart from structure and length, an important consideration often overlooked when describing predictions of functional compounds is the level of true novelty offered by the de-novo-discovered compounds. Accordingly, focusing our most promising candidate peptide, pep_1E99R5, presented in Table 1, we assessed the extent to which it was truly novel in light of (1) key human peptide hormones involved in glucose regulation and (2) the larger set of 1550 known endogenous human peptides (Figure 6B). To achieve this, we calculated three major peptide properties: charge, molecular weight and hydrophobicity. Here, pep_1E99R5′s charge (+1) and hydrophobicity (45.5%) are no different to most human endogenous peptides, with an average charge of +2.9 (50% of the peptides between −1 and +7) and an average hydrophobicity of 46.4 (50% of the peptides between 42.9% and 50.4%) (Figure S2). However, pep_1E99R5 (1.3 kDa) is substantially smaller than 99.6% of human endogenous peptides and smaller than both insulin (5.8 kDa) and GLP-1 (3.3 kDa), as well as on-the-market DM-treatment peptides Exenatide (4.1 kDa) and Liraglutide (3.7 kDa), which offers a considerable advantage in terms of production cost. pep_1E99R5 was further assessed against the natural glucose-regulating hormones in terms of homology, where, even at a very lenient e-value threshold, no sequence similarity was reported.

### 3.4. Molecular Mechanisms Modulated by pep_1E99R5

To elucidate the molecular pathways modulated by pep_1E99R5 in vitro, HSkMCs treated with the peptide underwent a full transcriptomic screen. HSkMCs treated with insulin were screened in parallel. To determine gene expression, RNA was extracted from the cells, fluorescently labelled and run in triplicate on SurePrint G3 Human Gene Expression v3 8 × 60 K Microarrays. Microarray technology simultaneously measures the expression of large numbers of transcripts in treated and untreated samples.

A 20-min stimulation of HSkMCs with 0.5 µg/mL (0.4 µM) pep_1E99R5 changed the expression, up or down (>1.3 fold; *p* < 0.01) of 625 transcripts, compared to untreated cells. A 1-µM insulin treatment of HSkMCs was run in parallel and changed the expression, up or down (>1.3 fold; *p* < 0.01) of 540 transcripts, compared to untreated cells (data not shown). KEGG analysis was used to obtain a biochemical overview of the pathways differentially expressed under the influence of pep_1E99R5 and insulin [59].

**Figure 7:**
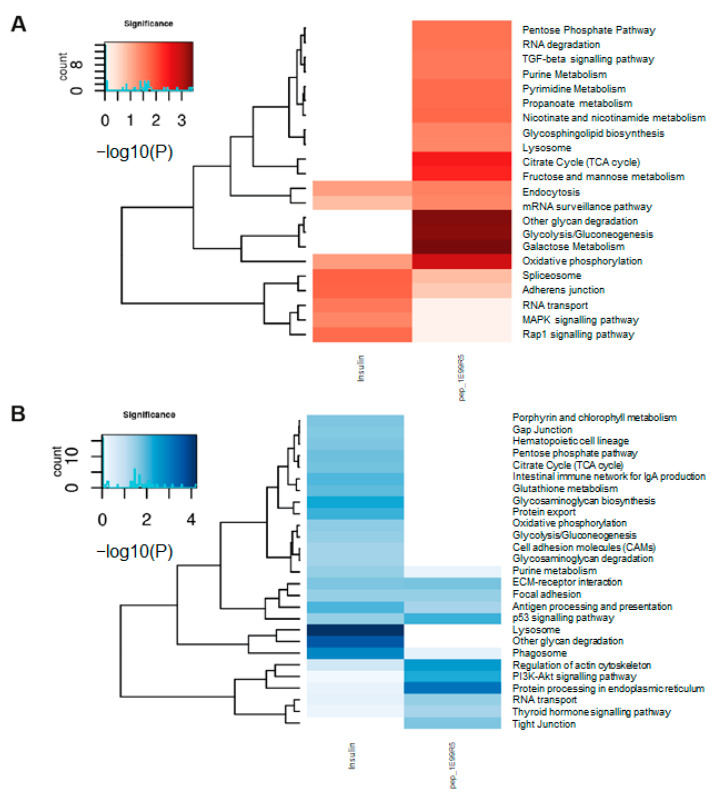
Kyoto Encyclopedia of Genes and Genomes (KEGG) pathway enrichment analyses of pep_1E99R5 and insulin. Representation of the KEGG pathway enrichment of significantly differentially expressed genes for insulin and pep_1E99R5 compared to untreated. Gene lists were filtered using raw *p*-value < 0.05 and fold change ≥ 1.3. The significance of a given KEGG pathway is assessed with raw *p*-value < 0.05. (**A**) Heatmap showing significantly downregulated (red) enrichment in KEGG pathways and (**B**) heatmap showing significantly upregulated (blue) enrichment in KEGG pathways (deeper colour indicates increased significance). Heatmaps present log-transformed raw *p*-values. Enrichment analyses were performed for each treatment (insulin and pep_1E99R5) separately and then brought together for comparison. Colour gradients indicate significance; the darker the colour, the more significant the result is.

Pathways related to glycolysis, oxidative phosphorylation and the citrate cycle were among the most highly ranked of the 21 KEGG pathways enriched by our peptide (all *p* < 0.01) (Figure 7). These pathways are typically downregulated in T2DM patients [60]. The pentose phosphate pathway, an alternative pathway to glycolysis, and the citrate cycle for oxidation of glucose also showed a trend toward enrichment when treated with pep_1E99R5, as did the TGF-β pathway, shown to stimulate the glucose uptake through GLUT1 [61]. Stimulation of these key glucose metabolism pathways with pep_1E99R5 may be key to its efficacy in enhancing glucose uptake in skeletal muscle cells. Nineteen KEGG pathways were downregulated by pep_1E99R5 (*p* < 0.01), including the PI3k-Akt pathway, the primary pathway for insulin-stimulated glucose uptake. This is further evidence that our peptide stimulates glucose uptake independent of the insulin pathway. The p53 signalling pathway was also downregulated by pep_1E99R5; typically, a tumour supressing pathway, this mechanism has the added ability to mediate metabolic changes in cells through the regulation of energy metabolism and has been shown to disrupt glucose uptake into cells [62]; hence, downregulation of this pathway will promote glucose uptake into cells.

The transcriptomic profile of cells treated with 1 µM insulin showed minimal KEGG pathway enrichment related to T2DM. This may be due to the relatively high insulin dose that the cells were exposed to, promoting upregulation in genes related to insulin resistance, including SOCS3, which was significantly upregulated by the insulin treatment and has been shown to be key to the physiological regulation of insulin signalling [63]. Conversely the KEGG pathway related to type I DM (T1DM) was among the pathways decreased by insulin treatment (*p* < 0.01). This finding is easier to interpret as the deficiency of bioavailable insulin is the primary mechanism by which T1DM arises. Furthermore, while the oxidative phosphorylation pathway showed a trend towards enrichment in the insulin-treated cells, this enrichment was less than that observed in the pep_1E99R5-treated samples. Comparison of significant gene enrichment in this pathway when the cells are stimulated with insulin (*n* = 3 genes) versus pep_1E99R5 (*n* = 9 genes) suggests that pep_1E99R5 has a greater effect in activating this key glucose metabolism pathway. While these data indicate different transcriptomic profiles for pep_1E99R5 and insulin, further work is underway to elucidate specific targets of pep_1E99R5.

Peptides represent the largest class of signalling molecules in animals, acting as hormones, neurotransmitters and growth factors to perform many critical physiological functions. Given that peptides have evolved to interact with specific biological targets, they offer great promise as selective, potent therapeutics that are less likely to suffer from issues of tolerability and toxicity [22,23]. Although beyond the scope of the current study, it is expected that natural peptides in their current form, due to their linear nature and absence of modifications, can circumvent issues with safety [64]. Indeed, no tolerability concerns were noted in any animals during both in vivo studies in this project with doses as high as 127 mg/kg.

To date, linear peptides as therapeutics have been largely underexplored. By their nature, they usually have an excellent safety profile and are easier to manufacture, with reduced loss of yield during synthesis [65]. Given their simple structure, a linear peptide can, in many cases, be optimised for bioavailability and stability more easily [42] than more structured, “difficult” peptides where optimisation alters efficacy [27,66,67]. Our aim here was to find a novel short natural linear peptide that can improve glucose modulation in vivo. When taking molecule size into account in current T2DM therapeutics, pep_1E99R5 is demonstrably smaller than current peptide therapeutics, which creates the potential for improved precision at the target site and reduced manufacture scale-up costs [68]. This coupled with the reduced economic burden observed following improved glycaemic control in T2DM of up to 13% [69], indicates further potential for such a peptide.

Understanding the mechanism of action (MOA) of pep_1E99R5 might reveal new mechanisms of glucose regulation in vivo; our results indicate that its MOA is different to that of insulin and further work is underway to elucidate this. Our initial study is a first step in investigating the world of natural linear peptides which, combined with good stability and bioavailability profiles, could become a repository for future therapeutic development.

The exploration of a diverse class of linear peptides and their association with glucose metabolism could not have been possible without the use of AI and machine learning. The predicted peptide, pep_1E99R5, is capable of modulating GLUT4 translocation, thereby affecting glucose uptake in vitro. Preclinical studies suggest that this peptide is biologically functional, leading to potential clinically relevant changes in both blood glucose and glycated haemoglobin, as well as a concomitant reduction in hepatic steatosis. Furthermore, analysis of the peptide itself, along with KEGG pathway analysis compared to insulin, suggests a unique, novel function of pep_1E99R5 in modulating blood glucose metabolism. An interesting application would be to integrate these machine learning approaches to explore the bioavailability and stability of linear peptides, which could give rise to candidates with not only good safety and efficacy profiles but also desirable pharmacokinetic properties for future therapeutic development in metabolic disorders such as T2D or others.

## 4. Conclusions

In undertaking this study, we aimed to explore short linear peptides with glucose-regulating activity and present experimental evidence that machine learning methods can reveal truly novel molecules capable of demonstrating meaningful and clinically relevant biological effects—in this case, in the context of T2DM. Of note, efficacious short linear peptides with good tolerability in vivo also present an opportunity for the pharmaceutical industry, with reduced manufacturing costs. Although further work is required to elucidate bioavailability, mechanism of action and clinical efficacy, we show initial evidence that unoptimised predicted peptides can display enhanced bioactivity in vitro than insulin and outperform Liraglutide in a hyperinsulinemic in vivo model. Ultimately, we highlight the capabilities of AI in discovery and present pep_1E99R5 as a short, linear bioactive peptide capable of affecting blood glucose metabolism in vitro and in vivo via robust modulation of a unique network of several key signalling pathways.

## Figures and Tables

**Table 1:** pep_1E99R5 characteristics.

Sequence	Length (Amino Acid)	Molecular Weight (Da)	Charge	Isoelectric Point	Hydrophobicity
WKDEAGKPLVK	11	1270.48	1	8.5	45.5%

## Data Availability

Publicly available datasets were analyzed in this study. This data can be found here (https://www.uniprot.org/statistics/Swiss-Prot).

## Supplementary Materials

The following are available online at https://www.mdpi.com/2227-9059/9/3/276/s1, Figure S1: AI predicted peptides in a mouse model of type 2 diabetes, Figure S2: Property dispersion of AI predicted peptides relative to known endogenous human peptides.
