# Supplementary material for: Discovery through Machine Learning and Preclinical Validation of Novel Anti-Diabetic Peptides
Source: Biomedicines. 2021 Mar 9;9(3):276. doi: 10.3390/biomedicines9030276 (PMC8000967; doi:10.3390/biomedicines9030276)
Supplement: Supplementary file 1 [file biomedicines-09-00276-s001.pdf]

# Discovery through Machine Learning and Preclinical Validation of Novel Anti-Diabetic Peptides

**Rory Casey[1], Alessandro Adelfio[1], Martin Connolly[1], Audrey Wall[1*], Ian Holyer[1] and Nora Khaldi[1]**

[1] Nuritas Ltd, Joshua Dawson House, Dublin 2, D02 RY95, Ireland
* Correspondence: **Author:** Dr Audrey Wall, PhD ; **Email:** wall.audrey@nuritas.com; **Phone:** +353 1 430 1290

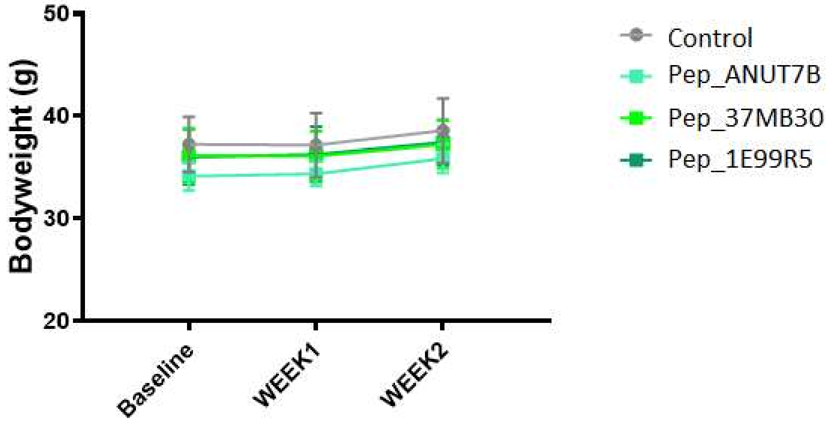


**Figure S1: AI predicted peptides in a mouse model of type 2 diabetes.** Bodyweight (g) of all mice were measured as indicated. Data are mean ± SEM (n=8 per group; aged 12 weeks at baseline) and analysed by Tukeys's test to compare the differences of bodyweight within each treatment group over the course of treatment (*p<0.05 **p<0.01 ***p<0.001)

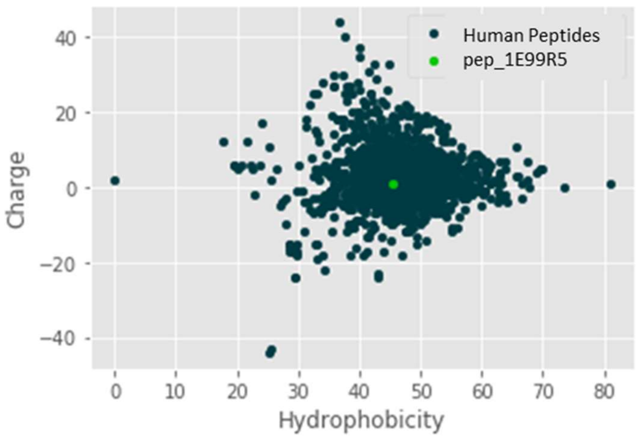


**Figure S2. Property dispersion of AI predicted peptides relative to known endogenous human peptides.**
